# Coliform Bacteria for Bioremediation of Waste Hydrocarbons

**DOI:** 10.1155/2017/1838072

**Published:** 2017-09-10

**Authors:** Majida Khanafer, Husain Al-Awadhi, Samir Radwan

**Affiliations:** Microbiology Program, Department of Biological Sciences, Faculty of Science, Kuwait University, P.O. Box 5969, 13060 Safat, Kuwait

## Abstract

Raw, domestic sewage of Kuwait City contained about 10^6^ ml^−1^ colony forming units of* Enterobacter hormaechei* subsp.* oharae* (56.6%),* Klebsiella* spp. (36%), and* Escherichia coli* (7.4%), as characterized by their 16S rRNA-gene sequences. The isolated coliforms grew successfully on a mineral medium with crude oil vapor as a sole source of carbon and energy. Those strains also grew, albeit to different degrees, on individual *n*-alkanes with carbon chains between C_9_ and C_36_ and on the individual aromatic hydrocarbons, toluene, naphthalene, phenanthrene, and biphenyl as sole sources of carbon and energy. These results imply that coliforms, like other hydrocarbonoclastic microorganisms, oxidize hydrocarbons to the corresponding alcohols and then to aldehydes and fatty acids which are biodegraded by *β*-oxidation to acetyl CoA. The latter is a well-known key intermediate in cell material and energy production.* E. coli* cells grown in the presence of *n*-hexadecane (but not in its absence) exhibited typical intracellular hydrocarbon inclusions, as revealed by transmission electron microscopy. Raw sewage samples amended with crude oil, *n*-hexadecane, or phenanthrene lost these hydrocarbons gradually with time. Meanwhile, the numbers of total and individual coliforms, particularly* Enterobacter*, increased. It was concluded that coliform bacteria in domestic sewage, probably in other environmental materials too, are effective hydrocarbon-biodegrading microorganisms.

## 1. Introduction

Environmental pollution with spilled oil and waste hydrocarbons has become a problem of major concern on the global scale. Those compounds contaminate water and terrestrial and atmospheric ecosystems. Early reports estimated that about 0.08 to 0.4% of the internationally produced crude oil is spilled into the environment [1]. Probably this value has currently increased because of the ever increasing activities related to oil extraction from reservoirs, transportation, processing, use as an energy source, and accidents and military conflicts. Spilled oil may be removed using physiochemical or microbiological means. The former approach comprises the use of sorbents [2, 3], solidifiers [4, 5], dispersants [6, 7], and gelators [8, 9]. However, the use of these products is not always environmentally safe and may even cause additional pollution [1, 10]. Bioremediation is the alternative approach commonly accepted as the most cost-effective and eco-friendly biotechnology available. Two practices are suggested for bioremediation: bioaugmentation and biostimulation [11]. In the former approach, oil-contaminated areas are inoculated with cocktails of hydrocarbonoclastic microorganisms [12–14]. Biostimulation, on the other hand, depends on enhancing already existing microorganisms by adding nutrients [11], surfactants [12, 15], and other materials. In this context, the characteristic feature of hydrocarbonoclastic microorganisms lies in their ability to utilize hydrocarbons as sole sources of carbon and energy through the well-established metabolic pathways. They possess hydroxylases (also called oxygenases) which introduce oxygen atoms from molecular oxygen into the hydrocarbon substrate producing fatty alcohols. The latter are further oxidized via the aldehydes (or ketones) to the corresponding fatty acids. The latter are biodegraded into the key intermediate metabolite acetyl CoA.

In bioaugmentation, an important question regarding the choice of hydrocarbonoclastic microorganisms to be inoculated should be considered. Essential prerequisites are that those organisms should easily succeed in colonizing the new environment and in standing competition with the already existing inhabitants [16, 17]. Therefore, the term “autochthonous bioaugmentation (ABA)” has been suggested recently for such a practice [18–20].

In the course of our work on bioremediation of desert and aquatic ecosystems that had been heavily polluted with oil during the greatest man-made oil spill at the end of the 1991 Gulf-war, we used to isolate hydrocarbonoclastic bacteria that according to their 16S rRNA-gene sequences were coliforms. Such bacteria belonged predominantly to the genera* Enterobacter*,* Klebsiella*,* Escherichia,* and others. The literature includes several reports by other investigators on hydrocarbon-degradation by such coliforms [21–23]. Even the hazardous simple and polyaromatic hydrocarbons were reported to be degraded by coliforms such as* Klebsiella* and* Enterobacter* [24–26]. Also diesel oil [27] and constituent hydrocarbons of crude oil [28–31] were found to be degraded by various coliforms. However, in many of those studies, the hydrocarbonoclastic potential was based solely on the ability of the strains to grow in mineral media with hydrocarbons as sole sources of carbon and energy. In some of those reports, the coliform taxa were just listed in tables including the identified hydrocarbonoclastic isolates, without further elaboration on criteria for considering them as hydrocarbon degraders.

Coliform bacteria belong to the family Enterobacteriaceae, which comprises facultative anaerobic bacteria naturally inhabiting digestive tracts of warm-blooded animals including man. Therefore, such microorganisms are continuously discharged with feces, and* E. coli*, for example, makes up about 10% of the domestic sewage microflora worldwide.

The major objective of this study was to investigate the hydrocarbonoclastic potential of coliforms from local domestic sewage samples. For this, microbiological, biochemical, and cytological techniques of analysis were integrated to obtain a more comprehensive evidence for the hydrocarbonoclastic potential of the obtained isolates. Should coliforms prove to be effective oil-biodegraders, domestic sewage would be a cost-effective, accessible source of microorganisms to be used in oil-bioremediation via bioaugmentation. Within this context, domestic sewage frequently receives waste hydrocarbons as constituents of disposed plant and animal wastes.

## 2. Materials and Methods

### 2.1. Counting and Characterization of Coliforms in Raw Sewage

Three local raw domestic sewage samples were collected from Al-Jahra station and used to count and isolate their indigenous coliforms. The conventional dilution-plating method was adopted, and the coliform-selective medium, Eosin Methylene Blue Agar (EMB) [32, 33], was used. The coliform colonies were counted, subcultured, and purified on the above selective medium. Individual coliforms were also counted after they had been differentiated according to their characteristic morphologies on this medium;* Enterobacter* colonies were pink with dark centers,* Klebsiella* colonies mucoid pink, and* Escherichia coli* colonies black with green metallic sheen The isolates were further characterized according to their potential for acid and gas production from lactose fermentation at 37°C and 44°C, following established routine procedures. Representative strains were isolated, purified, and characterized by comparing the sequences of their 16S rRNA-genes with sequences of type strains in the GenBank database. The method was essentially as follows: The total genomic DNA was extracted with PrepMan Ultra Sample Preparation Reagent (Applied Biosystems, USA) following the manufacturer's description. The 16S rRNA-genes were amplified by polymerase chain reaction (PCR) using a Veriti Thermal Cycler (Applied Biosystems, USA) and a standard protocol with initial denaturation at 95°C for 5 min and 32 cycles of denaturation at 94°C for 1 min, annealing at 55°C for 1 min, and primer extension at 72°C for 1 min. The mixture PCR contained puReTaq Ready-To-Go PCR Beads (Amersham Biosciences, UK), 25 ng of DNA template, 1 *μ*L of each of the universal primer combinations GM5F (5′-CCTACGGGAGGCAGCAG-3′) and 907R (5′-CCGTCAATTCMTTTGAGTTT-3′) [34]. The reaction volume was completed to 25 *μ*L with molecular water (Sigma, UK). Partial sequencing of the 16S rRNA-genes was done using a BigDye version Terminator Kit (Applied Biosystems, USA). The mixture contained 20 ng of the DNA template, 2 *µ*l of the BigDye version 3.1 terminator, 2 *µ*l of the 5x sequencing buffer, l *μ*L of either 907R, or GM5F and the final volume was brought up to 10 *μ*L with molecular water. Labeling was completed in the Veriti Thermal Cycler (Applied Biosystems, USA) using one cycle of 96°C for l min then 25 cycles of l min at 96°C, 5 s at 50°C, and 4 min at 60°C. The pure template DNA samples were processed in a 3130xl genetic analyzer (Applied Biosystems, USA). Sequencing analysis version 5.2 software (Applied Biosystems, USA) was used to analyze the results. Sequences were subjected to basic local alignment search tool analysis with the National Center for Biotechnology Information (NCBI, Bethesda, MD, USA) GenBank database [35]. A phylogenetic tree was constructed using neighbor joining including bootstrap analysis using PAUP^*∗*^ v.4 [36]. Bootstrap proportions were used on 2000 replicates.

### 2.2. Hydrocarbon Utilization Potential of Coliforms

All the coliform strains were tested for their ability to grow on a mineral medium [37] with oil vapor as a sole source of carbon and energy. The mineral medium had the following composition (gl^−1^): 5.0 NaNO_3_, 0.56 KH_2_PO_4_, 0.86 Na_2_HPO_4_, 0.17 K_2_SO_4_, 0.37 MgSO_4_·7H_2_O, 0.007 CaCl_2_·H_2_O, and 25 ml l^−1^ of a trace element solution consisting of (g l^−1^) 2.32 CuSO_4_·5H_2_O, 0.39 Na_2_MoO_4_·2H_2_O, 0.66 KI, 1.0 EDTA, 0.4 FeSO_4_·7H_2_O, and 0.004 NiCl_2_·6H_2_O. The pH was adjusted to 7. Strains capable of growth on that medium were considered “presumptive” hydrocarbonoclastic.

To study the range of pure hydrocarbons that could be utilized by the individual strains, a common inoculum (loopful in 5 ml sterile water) was prepared for each strain, and one-loopful aliquots were streaked on the solid mineral medium containing 0.5% of individual *n*-alkanes ranging in chain lengths between C_9_–C_20_, C_23_–C_26_, C_28_, C_30_, and C_36_. The aromatics toluene, naphthalene, phenanthrene, and biphenyl were also investigated. Cultures were incubated at 30°C for 15 d and examined for growth.

### 2.3. Hydrocarbon Consumption by Coliforms

The crude oil and pure-hydrocarbon consumption by individual isolates was measured quantitatively by gas liquid chromatography (GLC). Aliquots, 100 ml, of the mineral medium [37] in 250 ml flasks were supplied with 0.3% (w/v) crude oil or pure hydrocarbons. Each flask was inoculated with 1 ml cell suspension (a loopful of 48 h biomass in 5 ml sterile water). The cultures were sealed to avoid hydrocarbon losses by volatilization and incubated at 30°C on an electrical shaker, 200 rpm, for 2 weeks. Three parallel replicates were prepared throughout. The residual hydrocarbons were recovered with three successive 10 ml portions of pentane. The combined extracts were completed to 35 ml using pentane, and 1.0 *μ*l was analyzed by GLC. The percent loss of the peak areas based on the peak areas of the control samples (similarly prepared, but using previously autoclaved bacteria) was calculated as a quantitative measure of the oil-removal. For GLC, we used an Agilent 7890A GC (USA) system equipped with FID, a DB-5 capillary column (Agilent Technologies, USA), and He as a carrier gas. The oven temperature started at 50°C for 3 min, then rising at 3°C/min to 80°C, then rising at 8°C/min to 256°C, then rising at 30°C/min to 330°C, and holding at this temperature for 11 min.

### 2.4. Hydrocarbon-Uptake by Coliforms

In this experiment, two strains of* E. coli*, one from domestic sewage and the other from wheat straw [38], were investigated. Using transmission electron microscopy (TEM), we examined sections of cells previously grown in conventional nutrient broth and of cells grown in the mineral medium containing 0.3% *n*-hexadecane [37] for intracellular hydrocarbon inclusions. Biomass samples were fixed with 2.5% glutaraldehyde in sodium cacodylate buffer (0.1 M; pH 7.4) for 2 h [39]. The cells were washed in the same buffer, postfixed with 1.0% OsO_4_ (at 4°C for 2 h), and washed three times in the same buffer. Cells were centrifuged at 5,000 ×g for 10 min and the pellet was mixed with an equal volume of 2.0% noble agar. The agar was spread on a slide, left to solidify, and cut into small cubes. The cubes were dehydrated in an ascending series (30, 50, 70, and 100%) of acetone in water and rinsed twice in 100% propylene oxide. Finally, the samples were embedded in Epon resin. Ultrathin sections [40] were obtained using an ultramicrotome Leica (Leica Microsystems, Germany). The sections were mounted on carbon-coated grids, stained with saturated uranyl acetate and Reynolds lead citrate [41], and viewed in an electron microscope, JEM 1200-EX II (JEOL, USA).

### 2.5. Oil-Removal in Sewage Batches

Aliquots, 100 ml of freshly harvested raw sewage (not previously sterilized) in 250 ml flasks, were provided separately with 1.0 and 5.0 g portions of crude oil, 1.0 g portions of *n*-hexadecane, and 1.0 g portions of phenanthrene. The flasks were sealed to avoid hydrocarbon volatilization and incubated at 30°C on an electrical shaker, 200 rpm, for 20 d. At time zero and in 5 d intervals, cultures were harvested for quantitative oil and coliform analysis, as already described. Three replicate samples were analyzed throughout.

A similar parallel experiment was run but using previously autoclaved raw sewage aliquots, as controls.

### 2.6. Statistical Analysis

Three determinations for each analysis were done and the mean values ± standard deviation values were calculated using Microsoft Excel 2007. Also, Statistical Package for Social Sciences, version 12, was used to assess the degree of significance, where the analysis of variance (ANOVA) was used to differentiate between the means of the tested parameters.

## 3. Results and Discussion

### 3.1. Local Coliform Bacterial Strains

**Figure 1:**
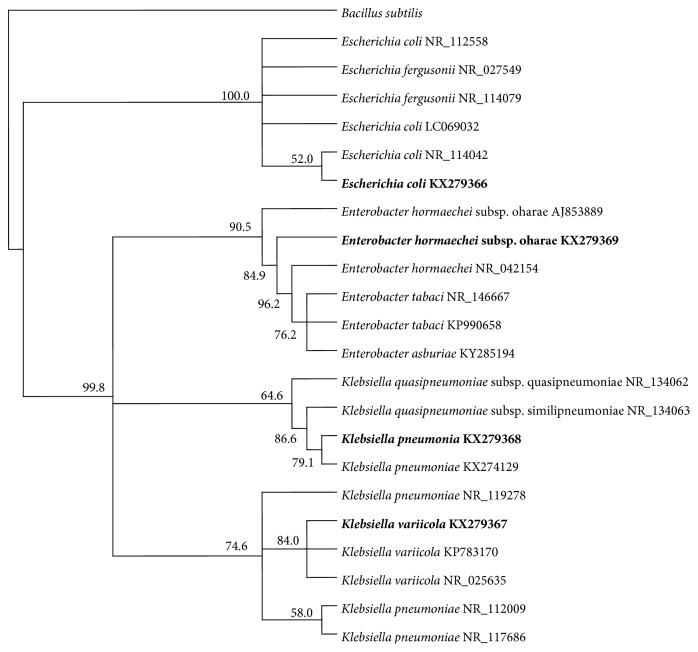
Phylogenetic tree showing relationships among coliforms isolated from domestic sewage.

The three raw sewage samples studied were found to contain 9.5 × 10^5^, 9.8 × 10^5^, and 9.6 × 10^5^ ml^−1^ colony forming units (CFU) of total coliform bacteria as counted on the selective coliform medium. Based on their 16S rRNA-gene sequences, those coliforms consisted of* Enterobacter hormaechei* subsp.* oharae*, 56–58%;* Klebsiella variicola*, 23–25%;* Klebsiella pneumonia*, 10-11%; and* Escherichia coli*, 7–10%. The results in Table 1 show that the sequence similarities between the isolates and the standard strains in the GenBank database were high, between 99 and 100%. The phylogenetic relationships among the isolates are shown in the phylogenetic tree in Figure 1. The results of the conventional test for acid and gas production from lactose by the 4 coliform isolates (Table 2 and Figure S1 in the Supplementary Material available online at https://doi.org/10.1155/2017/1838072) were those expected according to their generic identities.

### 3.2. Oil and Hydrocarbon Utilization by the Coliform Isolates

A total of 313 pooled coliform colonies (on the selective EMB medium) were subcultured and purified. All the pure cultures grew on a solid mineral medium [37] with crude oil vapor as a sole source of carbon and energy. This result indicates that all the isolated coliforms presumably have potential for hydrocarbon utilization.

Five haphazardly selected strains for each coliform species were found to grow, albeit with different intensities, on the solid mineral medium [37] supplied with the individual *n*-alkanes, C_9_–C_20_, C_23_–C_26_, C_28_, C_30_, and C_36_, and the individual aromatic hydrocarbons, toluene, naphthalene, phenanthrene, and biphenyl. The growth on such a wide range of aliphatic and aromatic hydrocarbons reflects a high potential for hydrocarbon utilization. Earlier investigators also found that the same coliform species isolated elsewhere could grow on oil and hydrocarbons as sole sources of carbon and energy [21–23]. Successful growth of all tested coliforms on aliphatic and aromatic hydrocarbons suggests that they, like all hydrocarbonoclastic microorganisms, produce monooxygenases and dioxygenases (hydroxylases), respectively [42–44]. Moreover, the further metabolic pathways in such coliforms are apparently similar to those in other hydrocarbonoclastic microorganisms. The fatty alcohols are oxidized to aldehydes (ketones) and ultimately to fatty acids, which in turn are degraded by *β*-oxidation to acetyl CoA units. The latter could then be used for synthesis of cell material and for the production of ATP through the tricarboxylic acid cycle.

The results in Table 3 show that representatives of the local coliform isolates removed quantitatively significant proportions of the available crude oil as well as of the pure aliphatic (C_16_) and aromatic (phenanthrene) hydrocarbons in 15 days. Those hydrocarbons were provided in the mineral medium [37] as sole sources of carbon and energy. These quantitative determinations provide a clear-cut evidence for the hydrocarbonoclastic potential of the studied coliforms.

### 3.3. Cytological Evidence for *n*-Hexadecane (C_16_) Uptake by* E. coli*

**Figure 2:**
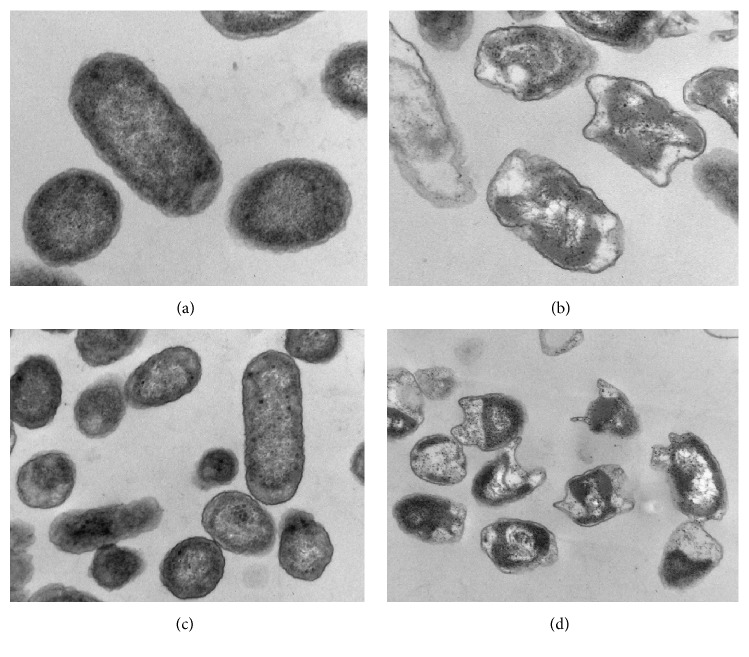
Transmission electron micrographs of ultrathin sections of* E*.* coli* cells from domestic sewage grown in *n*-hexadecane-free medium (a) and in *n*-hexadecane-containing medium (b) and of* E*.* coli* cells from wheat straw grown in *n*-hexadecane-free medium (c) and in *n*-hexadecane-containing medium (d). Magnification: (a) and (b) ×60000; (c) and (d) ×40000.

For this experiment, two strains of the typical coliform,* E. coli*, one from sewage and the other from wheat straw [38], were used. The TEM images in Figure 2 show that the cell cytoplasm of hydrocarbon-incubated cells, in contrast to that of cells harvested from nutrient broth (without hydrocarbons), was enriched with less electron-dense areas (hydrocarbon inclusions) of varying dimensions. Our measurements showed that those inclusions occupied roughly 50% of the total cell volumes. Earlier researchers also described similar intracytoplasmic hydrocarbon inclusions in classical hydrocarbonoclastic bacteria incubated in media containing *n*-hexadecane [37]. Cells incubated in the presence of C_16_ also showed clear morphological deformations.

### 3.4. Self-Cleaning of Oily Sewage by Indigenous Coliforms

**Figure 3:**
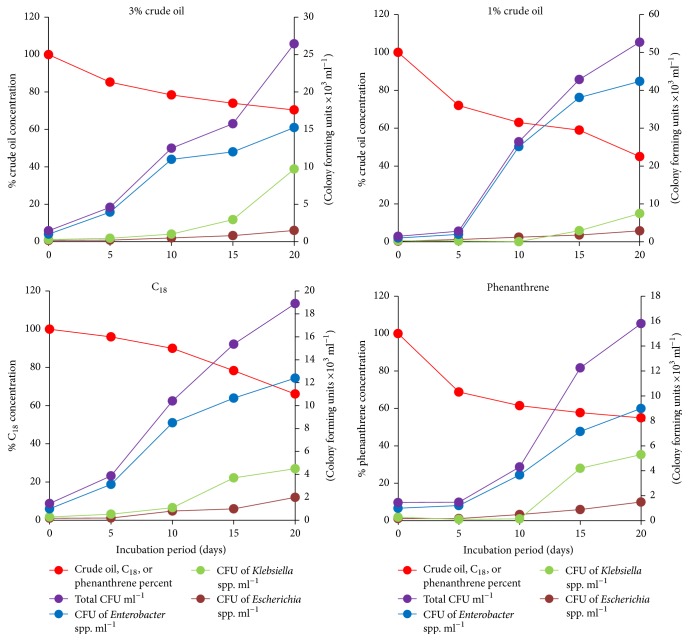
Consumption of crude oil added to freshly harvested sewage and parallel increase of total coliform members with time.

The results in Figure 3 show that crude oil, C18, and phenanthrene added to freshly harvested raw sewage (not previously sterilized) were steadily removed during the 20 d incubation period. At the end, the remaining proportions were only 70% of 3% crude oil, 45% of 1% crude oil, 66% of C18, and 55% of phenanthrene, based on the amount added at time zero. Meanwhile, there was a parallel increase of the total numbers of the total coliforms from 1.46 × 10^6^ to 26.5 × 10^6^ CFU ml^−1^ in 3% crude oil, to 52.7 × 10^6^ CFU ml^−1^ in 1% crude oil, to 18.9 × 10^6^ CFU ml^−1^ in C18, and to 15.8 × 10^6^ CFU ml^−1^ in phenanthrene. Figure 3 shows further that* Enterobacter* was the coliform that exhibited the highest increase in number, which may reflect a main role of this coliform in oil consumption. Other hydrocarbonoclastic, noncoliform bacteria indigenous to sewage are also probably involved in the measured crude oil removal. However, the significant increase (*p* < 0.05) in numbers of coliforms parallel to the significant decrease (*p* < 0.05) in the concentration of the remaining oil provides an experimental evidence for a major contribution of indigenous coliforms to self-cleaning of oil in sewage. This finding implies that the indigenous domestic sewage microflora is capable of controlling potential oil spills in this waste product. Furthermore, raw sewage is an accessible, cost-effective source of hydrocarbonoclastic microbial cocktails that can be used for bioaugmenting oil-contaminated areas.

## 4. Conclusions

The results of this paper provide experimental evidence for the role of coliforms in biodegradation of oil spilled in the environment. This group should be particularly effective in cleaning oil spills in sewage, where those organisms are autochthonous (indigenous). Conditions prevailing during conventional sewage treatment probably bioenhance the coliforms potential for oil-bioremediation. On one hand, sewage treatment involves the routine practice of extensive aeration to reduce the sewage BOD values (i.e., the organic matter content). In view of the fact that the initial step of microbial attack on hydrocarbons is catalyzed by oxygenases (also called hydroxylases), aeration should bioenhance the spilled-oil bioremediation. Oil-bioremediation is also bioenhanced by nitrogen-fertilizers. During sewage treatment, nitrates and other nitrogenous compounds are continuously produced and in addition several coliforms, for example,* Klebsiella* spp. and* Enterobacter* spp., are diazotrophic. In other words, nitrogen fertilization is also guaranteed in sewage for the hydrocarbonoclastic coliform communities.

## Supplementary Material

Fresh cultures of the isolated coliforms were inoculated into tubes containing lactose peptone water and Durham tubes. Two batches were prepared; one was incubated at 37°C and the other at 44°C for 24 h. The tubes were inspected for acid (yellow color) and gas (in the Durham tubes) production.

## Figures and Tables

**Table 1:** Information about the 16S rDNA sequencing of the coliforms isolated from domestic sewage.

Nearest GenBank match	Total bases	Similarity (%)	Compared bases	Accession number
*Escherichia coli*	513	100	513/513	KX279366
*Klebsiella variicola*	482	99	484/485	KX279367
*Klebsiella pneumoniae*	507	100	507/507	KX279368
*Enterobacter hormaechei* subsp. *oharae*	506	99	511/514	KX279369

**Table 2:** Acid and gas production from lactose by coliform isolates.

Isolates	Acid production		Gas production	
	37°C	44°C	37°C	44°C
*Escherichia coli* (wheat straw)	**+**	**+**	**+**	**+**
*Escherichia coli* (domestic sewage)	**+**	**+**	**+**	**+**
*Klebsiella variicola*	**+**	**+**	**+**	−
*Klebsiella pneumoniae*	**+**	**+**	**+**	−
*Enterobacter hormaechei* subsp. *oharae*	**+**	**+**	**+**	−

**Table 3:** Crude oil and pure-hydrocarbon consumption by coliform isolates.

Isolates	% hydrocarbon consumption		
	Crude oil	C16	Phenanthrene
*Escherichia coli* (wheat straw)	18.3 ± 1.1	17.1 ± 1.0	16.3 ± 0.9
*Escherichia coli* (domestic sewage)	22.2 ± 3.2	19.0 ± 2.2	18.1 ± 3.0
*Klebsiella variicola*	16.6 ± 1.0	12.3 ± 0.8	11.0 ± 0.5
*Klebsiella pneumoniae*	14.2 ± 1.2	10.6 ± 0.8	10.9 ± 1.0
*Enterobacter hormaechei* subsp. *oharae*	30.3 ± 1.5	35.9 ± 2.0	18.5 ± 0.8
*Pseudomonas stutzeri*	15.6 ± 0.9	18.4 ± 0.8	14.1 ± 0.7

*Pseudomonas stutzeri* from our private culture collection was analyzed as a classic hydrocarbon-utilizer for the purpose of comparison.
