# Supplementary material for: Coliform Bacteria for Bioremediation of Waste Hydrocarbons
Source: Biomed Res Int. 2017 Sep 10;2017:1838072. doi: 10.1155/2017/1838072 (PMC5610891; doi:10.1155/2017/1838072)
Supplement: Supplementary file 1 — Fresh cultures of the isolated coliforms were inoculated into tubes containing lactose peptone water and Durham tubes. Two batches were prepared; one was incubated at 37°C and the other at 44°C for 24 h. The tubes were inspected for acid (yellow color) and gas (in the Durham tubes) production. [file 1838072.f1.pdf]

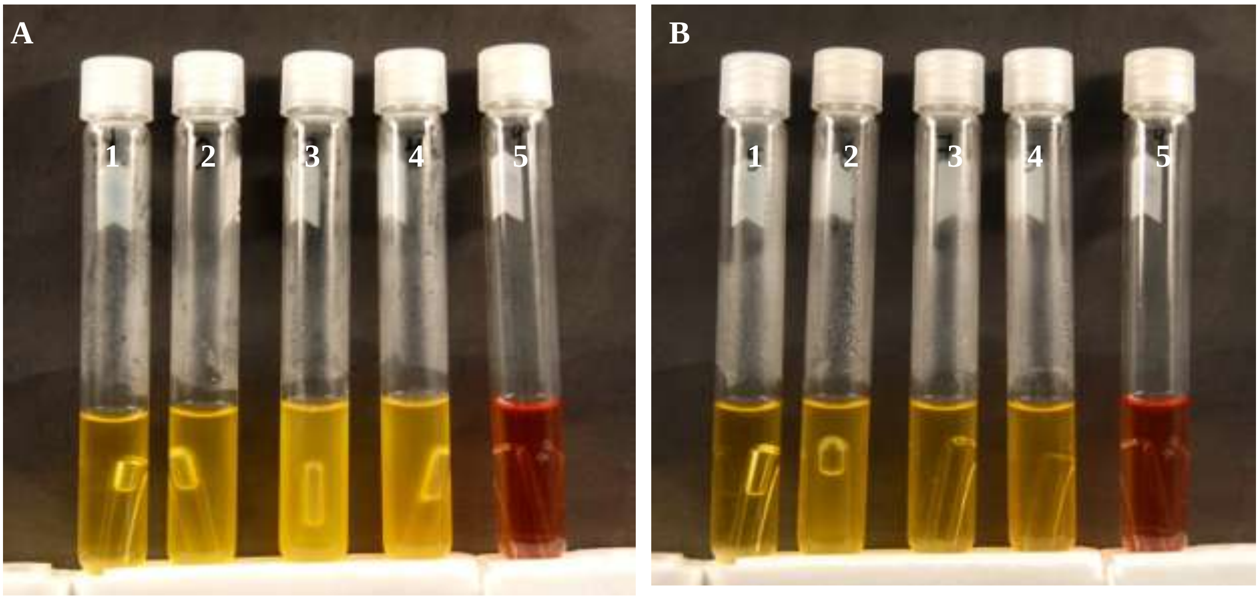

FIGURE S1: Acid and gas production from lactose by coliform isolates after 24 h incubation at 37 °C (A) and 44 °C (B).

Fresh cultures of the isolated coliforms were inoculated into tubes containing lactose peptone water and Durham tubes. Two batches were prepared; one was incubated at 37 °C and the other at 44 °C for 24h. The tubes were inspected for acid (yellow color) and gas (in the Durham tubes) production.

1, *Escherichia coli* (wheat straw); 2, *Escherichia coli* (domestic sewage); 3, *Klebsiella variicola*; 4, *Enterobacter hormaechei* sub sp. oharae; 5, control, no inoculum.
